# Further Uses of a Speedometer

**Published:** 1910-02-26

**Authors:** 


### FURTHER USES OF A SPEEDOMETER.

A great advantage of a speedometer is that it enables one to maintain the proper speed to reach a given destination on time without troublesome reference to the watch and odometer, or without maintaining an excessive speed. Let us suppose that we wish to reach a given point about 35 miles away in two hours. It is evident that this calls for an average speed of about 17 miles per hour. Let us say that the first part of the route, for about 20 miles, is over good, level, macadam roads, and that the last 15 miles are over hilly, poor roads, where, on account of the bad conditions, it is not wise to force the speed. Under these conditions a good driver will probably maintain, as nearly as possible, a speed of about 20 miles per hour for the first part of the way, and look at his watch probably at the end of each ten miles. Let us say that at the end of the first 20 miles 55 minutes have expired. A slight mental calculation will show that there is an hour and five minutes left to do the remaining 15 miles, or that the average rate need only be a little less than 15 miles per hour; thus the driver need not push his car over the poor roads.

Many agents nowadays fit their demonstration cars with speedometers, and a prospective purchaser may learn much of the qualities of a given car by watching the action of the instruments. The way a car will gain speed on the level is an indication of the liveliness of the engine or the power to accelerate under load. Again, the holding or gaining of speed on a hill is indicated by the speedometer. This is undoubtedly the real test of hill-climbing capability, and not the ability to surmount any given hill on the top gear. In climbing a hill note the speed at the bottom and then at the top. The car which falls off the least, gearing and size of road wheels being equal, shows the most staying powers. On the other hand, the slower the speed at the bottom, weights being equal, the more a given car may be expected to fall off, owing to its momentum being less. In testing the acceleration of a car, get the driver to put it, let us say, at 15 miles per hour, and at a signal to speed up the engine; note the time it takes to get to, say, 20 miles per hour. Due consideration being given to gearing, weight of car, size of wheels, etc., the acceleration and hill-climbing tests should be of great service in judging the qualities of a car.

